# Supplementary material for: An anti-inflammatory activation sequence governs macrophage transcriptional dynamics during tissue injury in zebrafish
Source: Nat Commun. 2022 Sep 20;13:5356. doi: 10.1038/s41467-022-33015-3 (PMC9489698; doi:10.1038/s41467-022-33015-3)
Supplement: Supplementary file 1 — Supplementary Information [file 41467_2022_33015_MOESM1_ESM.pdf]

Supplementary Information

An anti-inflammatory activation sequence governs macrophage transcriptional dynamics during tissue injury in zebrafish

Nicolas Denans[1]*, Nhung T. T. Tran[1], Madeleine E. Swall[1], Daniel C. Diaz[1,2], Jillian Blanck[1] and Tatjana Piotrowski[1]*

*Corresponding authors. Email: ndenans@stowers.org and pio@stowers.org

This PDF file includes:

Supplementary Figures 1 to 10

Other Supplementary Information:

Supplementary Movies 1 to 5
Supplementary Data 1 to 3

Supplementary Figure 1

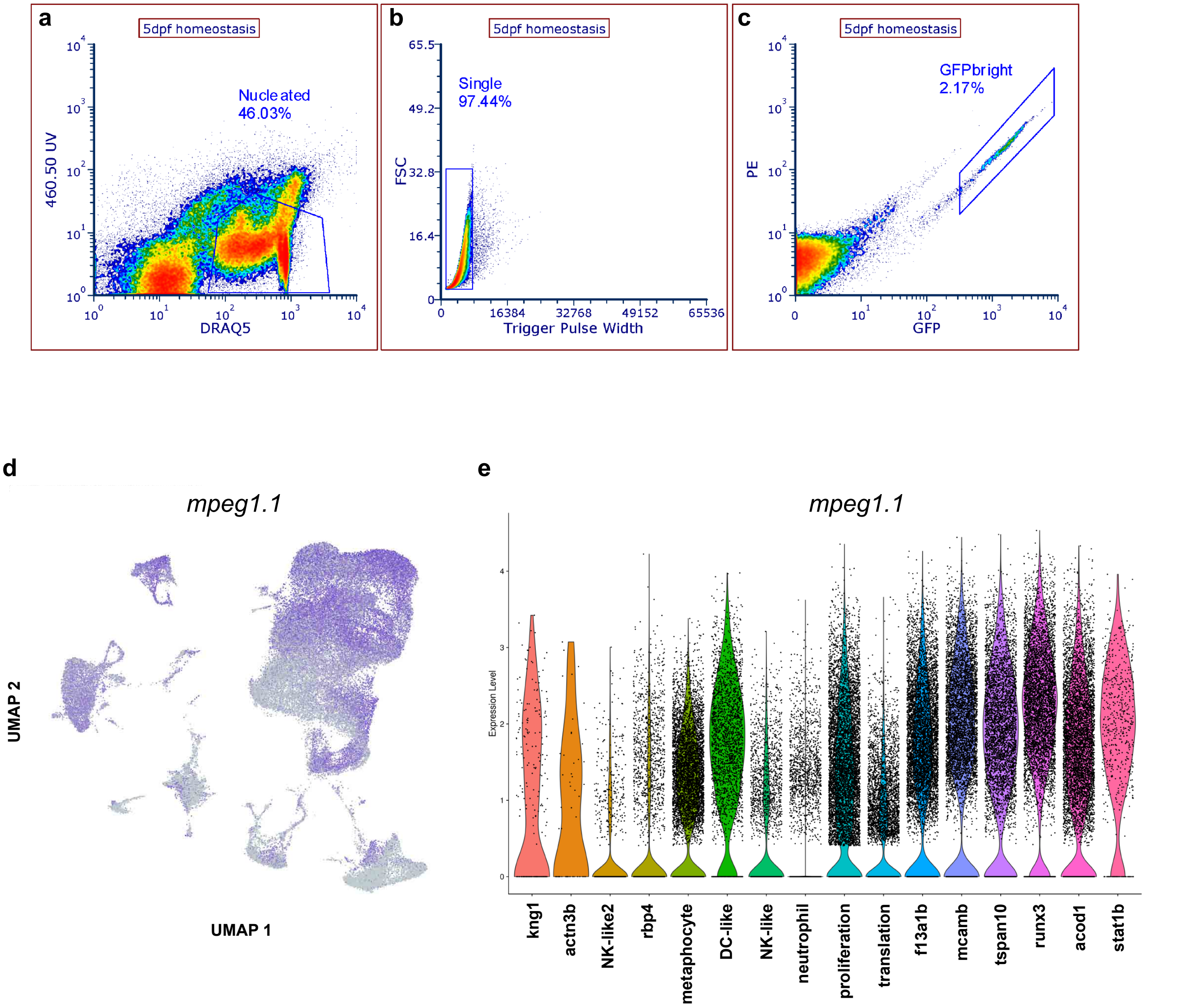

Supplementary Figure 1. *Tg(mpeg1:GFP)* labels several immune cell types and is not macrophage specific.

(a-c) FACS profile of GFP+ cells collected for scRNA-seq. **(d)** Feature plot for *mpeg1.1*. **(e)** Violin plot for *mpeg1.1*.

Supplementary Figure 2

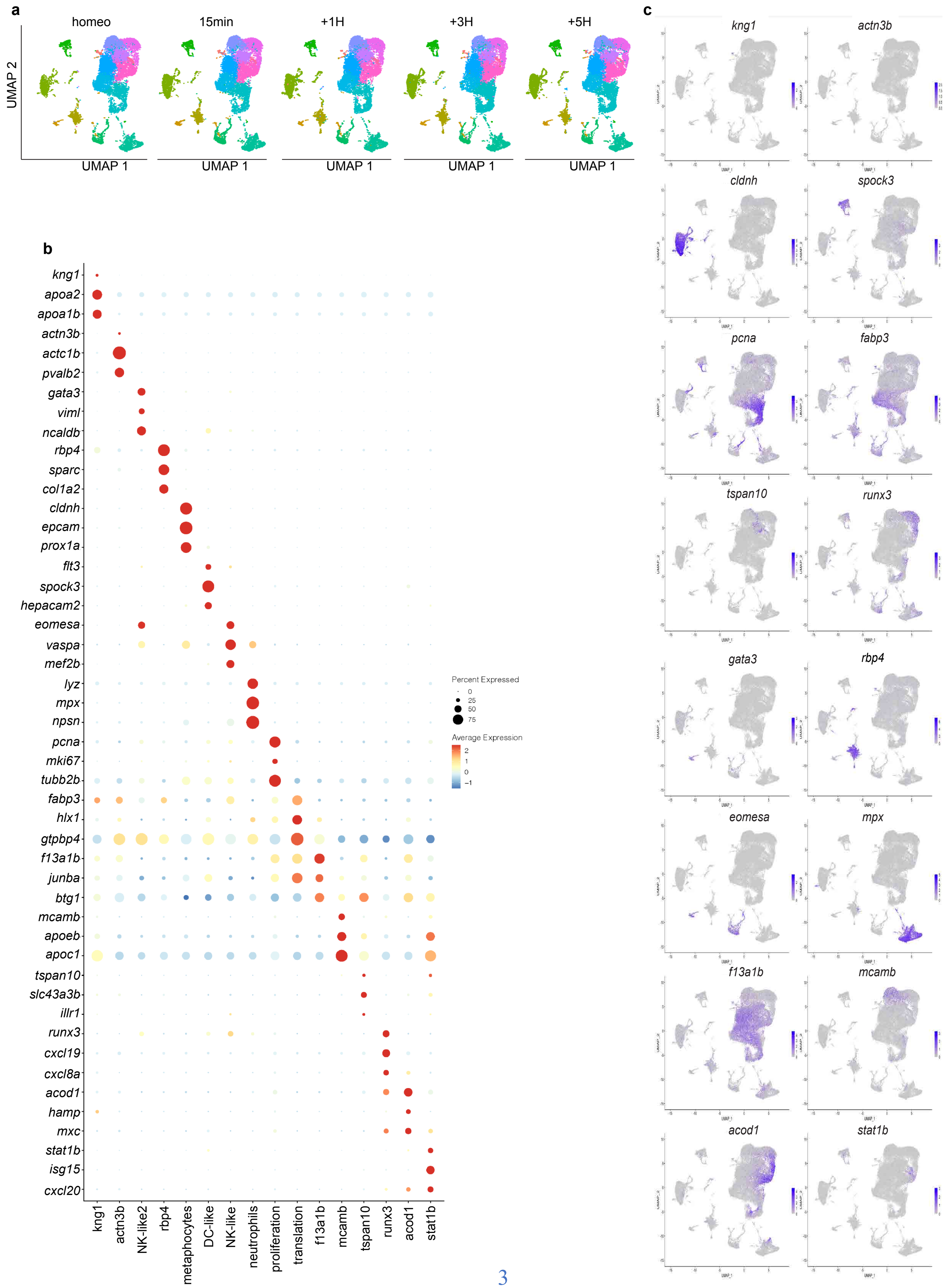

Supplementary Figure 2. Cluster markers from the macrophage scRNA-seq time course. (a) Individual UMAP for each dataset (time point). 14000 cells per dataset. (**b**) DotPlot showing three marker genes per cluster. (**c**) Feature plots of cluster marker genes.

Supplementary Figure 3

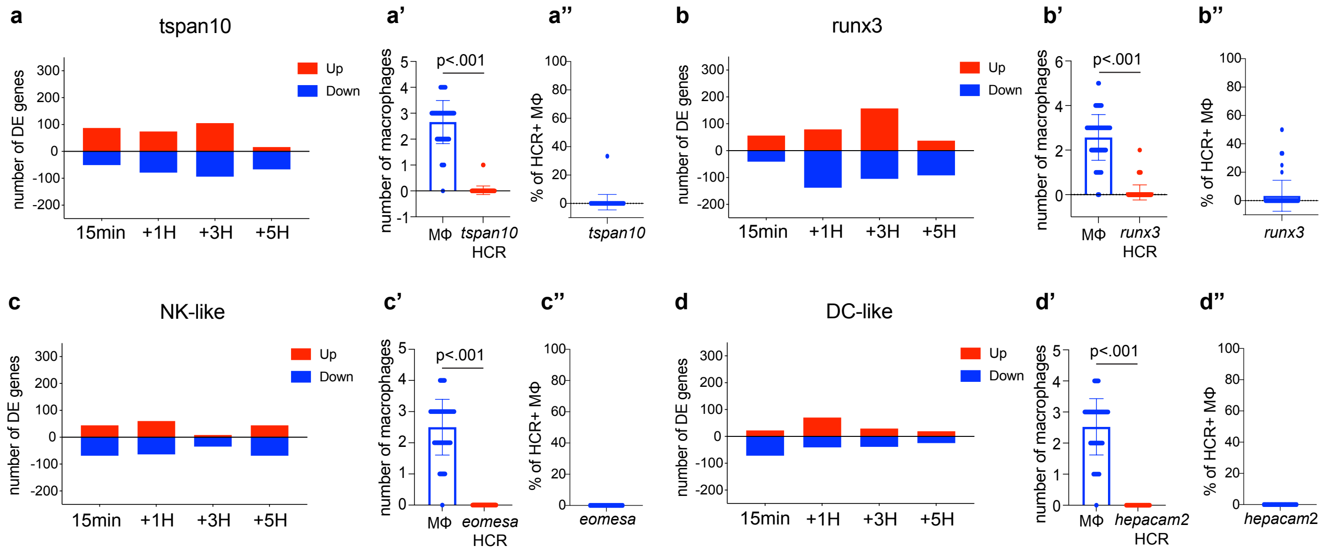

Supplementary Figure 3. Even non-effector macrophages show gene expression changes. (a, b, c and d) Quantification of differentially expressed genes. Upregulated genes in red, downregulated genes in blue at each time point. (**a’, b’, c’ and d’**) Quantifications of GFP+ effector macrophages (MΦ) and effector macrophages with a positive HCR signal. Each dot represents the number of macrophages per neuromast (5 neuromasts per larvae, 16 larvae and 3 biological replicates). P-values represent non-parametric two-tailed Student’s t-test. (**a”,b’’, c” and d’’**) Quantifications of the percentage of HCR+ effector macrophages. Each dot represents the number of macrophages per neuromast (5 neuromasts per larvae, 16 larvae and 3 biological replicates). For all graphs, data are represented as mean +/- SD.

Supplementary Figure 4

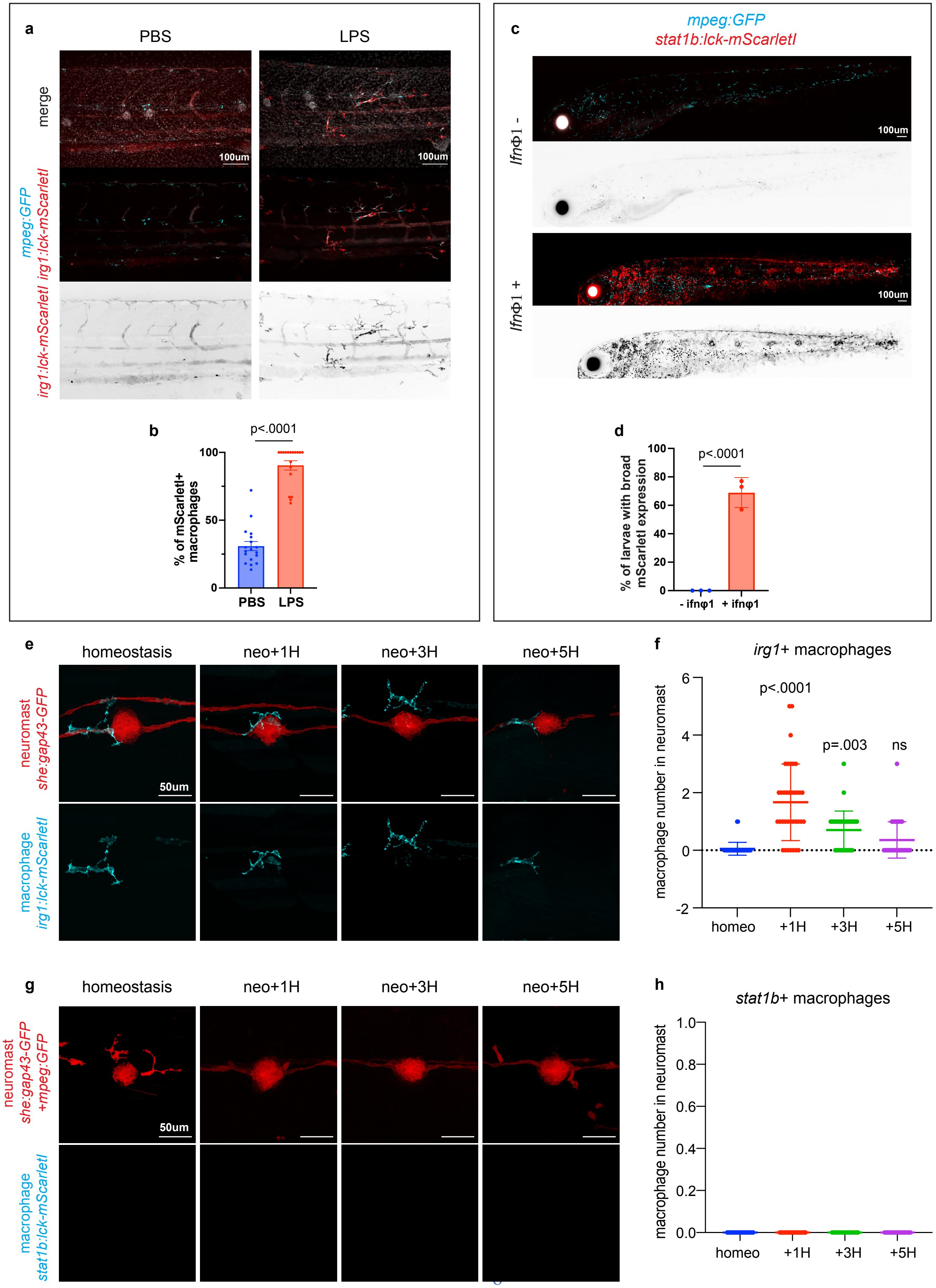

Supplementary Figure 4. ‘irg1/acod1’ but not ‘stat1b’ macrophages are effector cells. (a) Representative confocal images (projection of 150µm z-stack) of *tg(-5.6irg1:lckmScarletI)* larvae injected with either PBS or LPS**. (b)** Quantification of the percentage of mScarletI positive macrophages. Each dots represents 1 larva and is the result of 3 independent experiments. P-value represent a two-tailed Student’s t-test. **(c)** Representative confocal images (projection of 200µm z-stack) of *tg(-9stat1b:lckmScarletI)* larvae injected with *infphi1* or uninjected. **(d)** Quantification of the percentage of larvae with broad mScarletI expression. Each dots represents 1 independent experiment. P-value represents a two-tailed Student’s t-test. **(e, g)** Representative confocal images (projection of a 30µm z-stack) of the macrophage recruitment assay. (**f, h**) Quantification of macrophages inside the neuromasts. Each dot represents the number of macrophages per neuromast (3 neuromasts per larvae and 6 larvae per condition and 3 independent experiments). A 2-way ANOVA followed by a Tukey multiple comparison test has been used to determine statistical significance. P-values represent a post-hoc (Tukey) test from each condition relative to homeostasis. For all graphs, data are represented as mean +/- SD.

Supplementary Figure 5

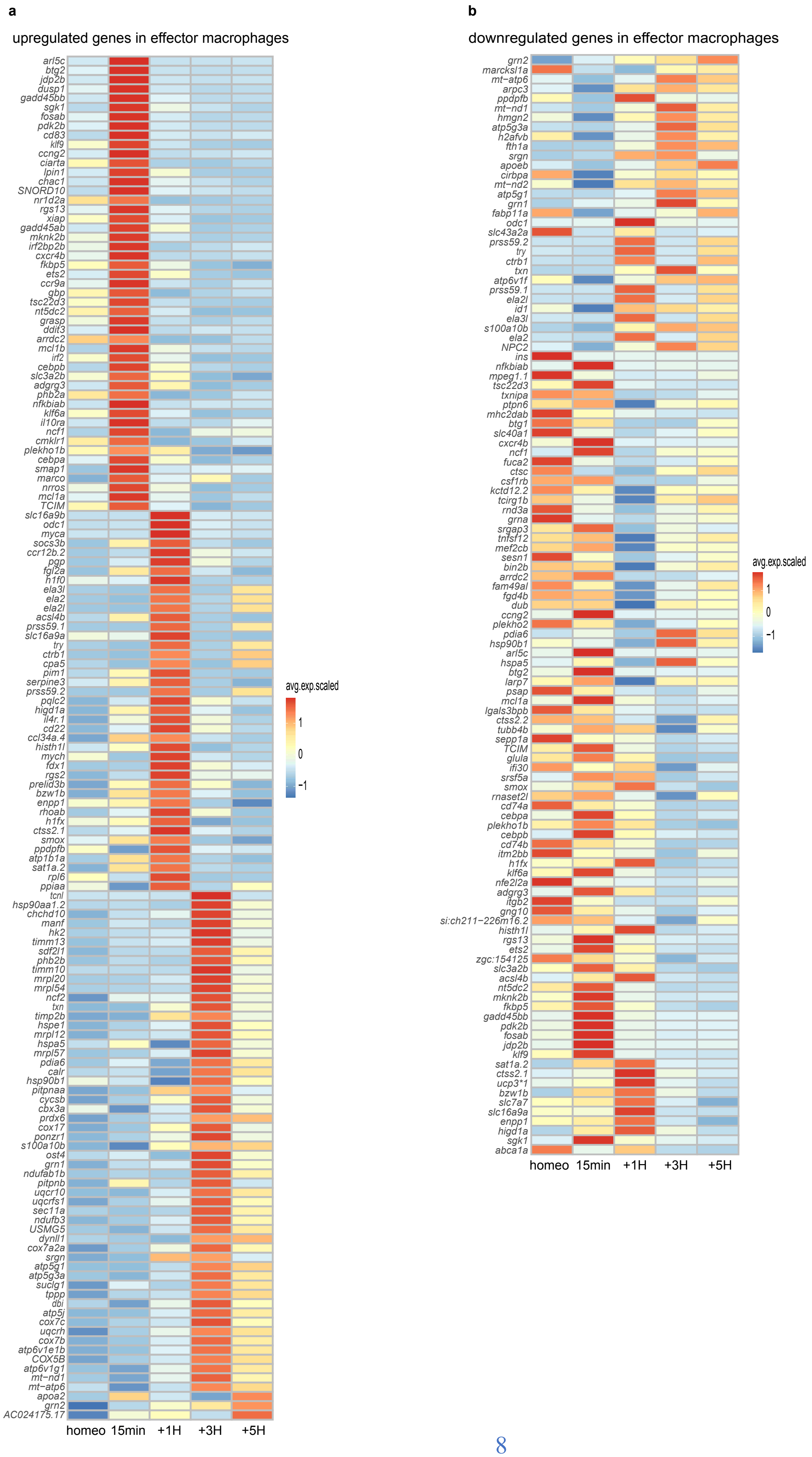

Supplementary Figure 5. Differentially expressed genes at each time point. (a-b) Heatmaps for (a) up- and (b) downregulated genes at each time point.

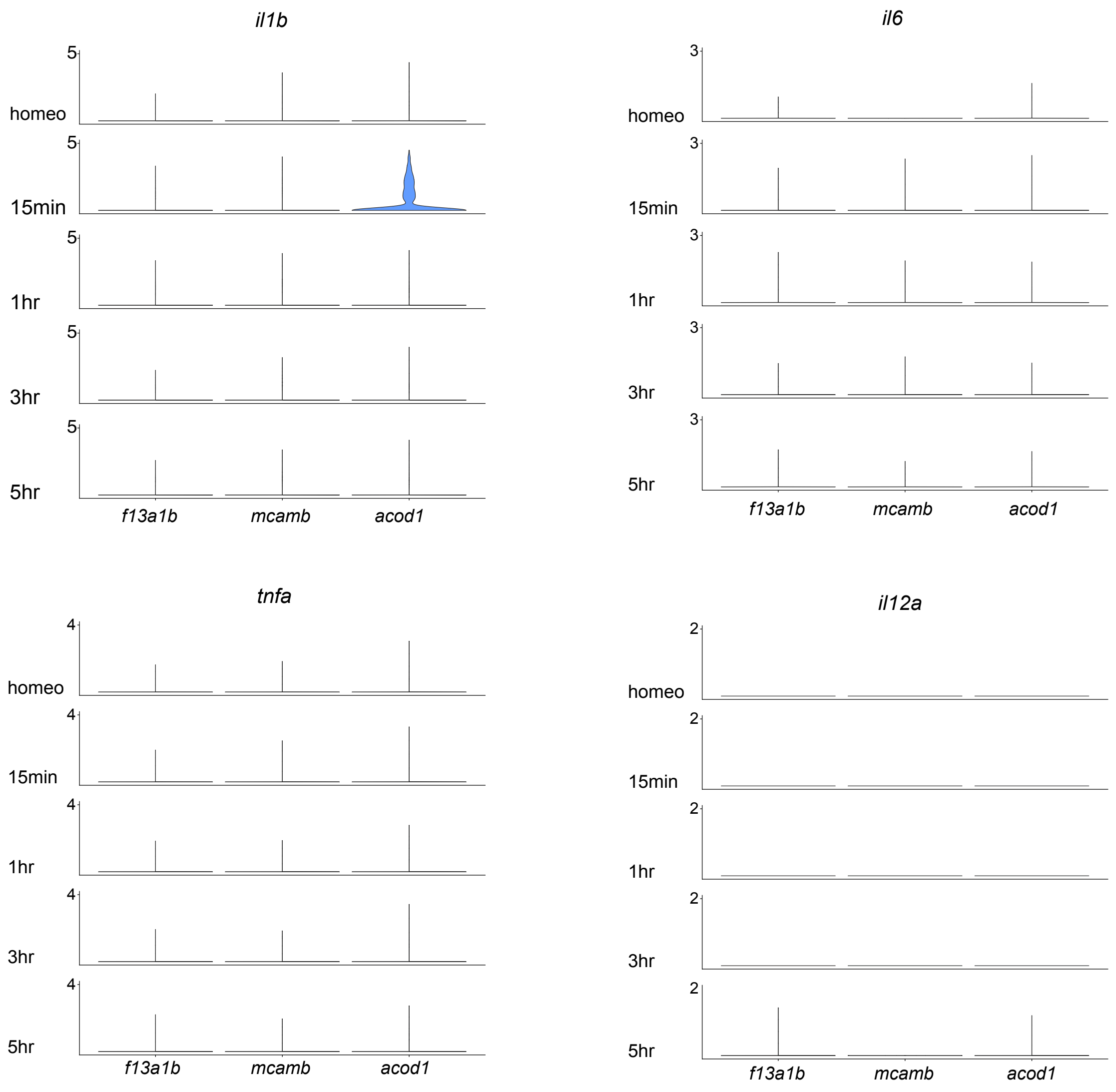

Supplementary Figure 6. Most pro-inflammatory cytokines are not transcriptionally upregulated in effector macrophages after HC death. Stacked Violin-Plots for *il1b*, *il6*, *tnfa* and *il12a.*

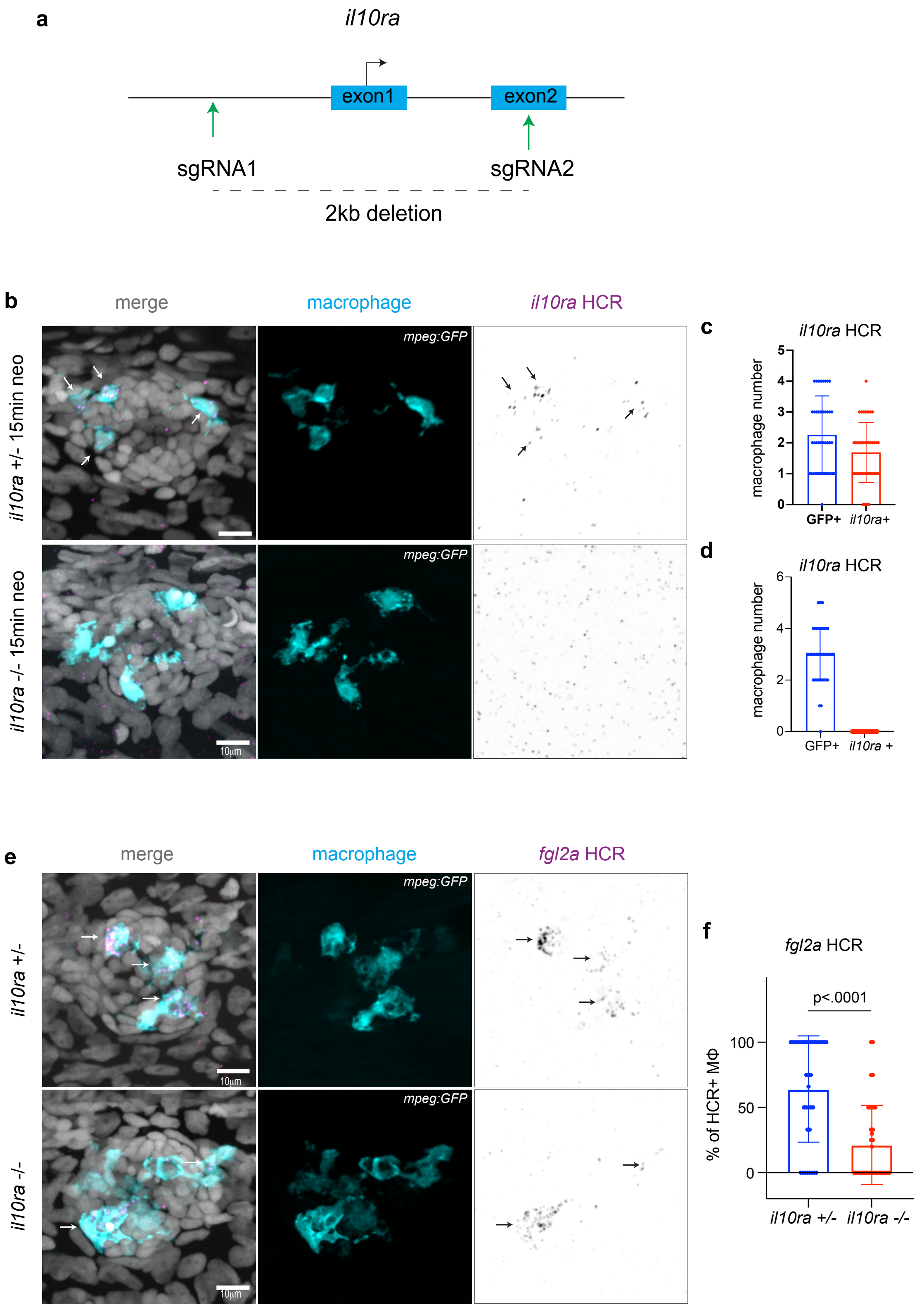

Supplementary Figure 7. Validation of the *il10ra* mutant. (a) Schematic depicting the location of the 2kb deletion in the *il10ra* locus. **(b)** Representative confocal images (projection of a 30µm z-stack) of HCR-FISH within the effector macrophages with *il10ra* (arrows)**. (c-d)** Quantification of the number of GFP+ cells positive for *il10ra* HCR in (c) *il10ra+/-* (n=42 neuromasts from 14 larvae in 3 biological replicates) and (d) *il10ra-/-* (n=48 neuromasts from 16 larvae in 3 biological replicates) **(e)** Representative confocal images (projection of a

30μm z-stack) of HCR-FISH within the effector macrophages for *fgl2a* (arrows). (**f**) Quantifications of the percentage of HCR+ effector macrophages for *fgl2a* in *il10ra* mutant heterozygous and homozygous larvae (n=36 neuromasts from 12 larvae per condition, 3 biological replicates). P-values represent non-parametric two-tailed Student's t-test. For all graphs, data are represented as mean +/- SD.

Supplementary Figure 8

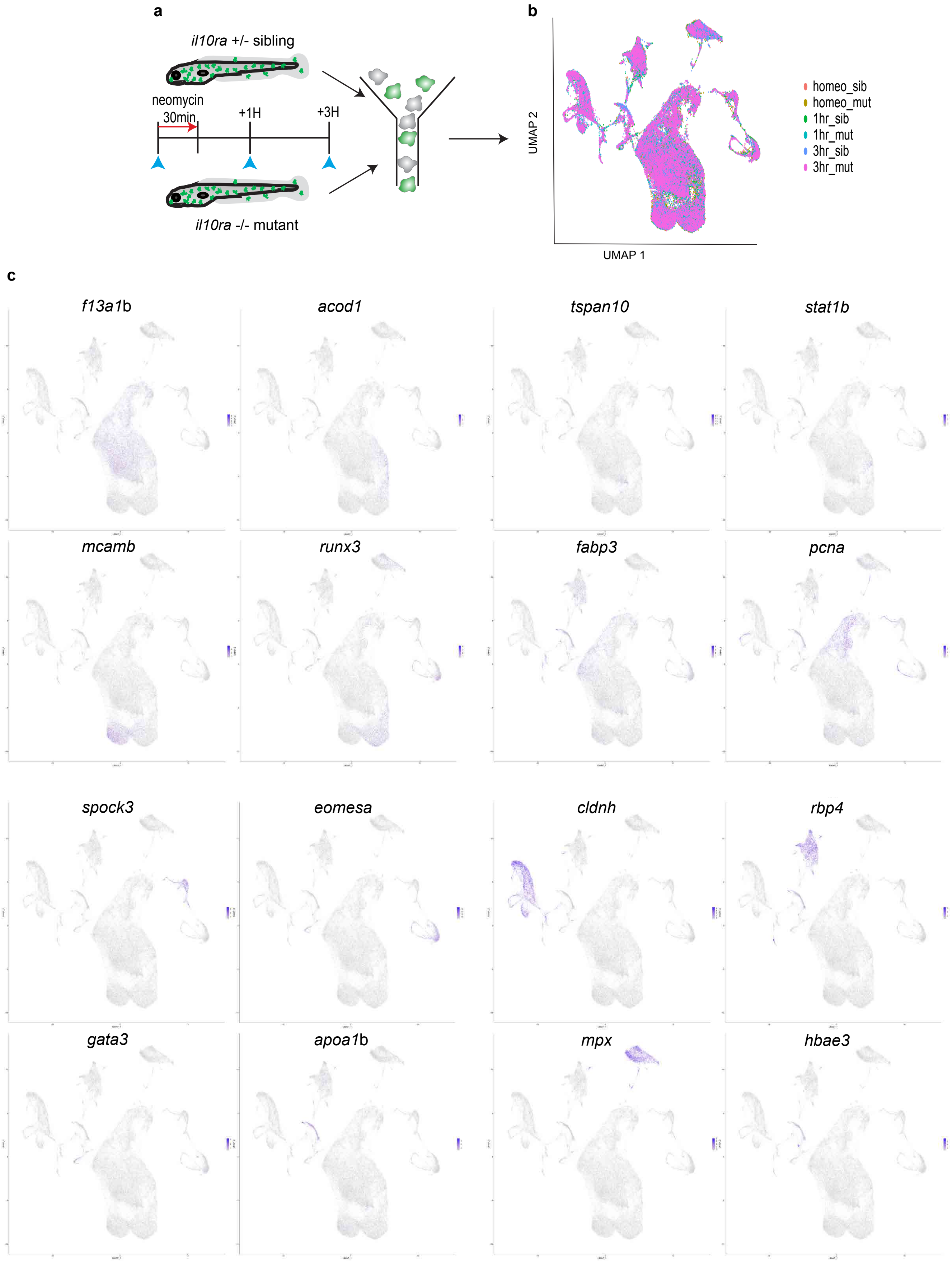

Supplementary Figure 8. Cluster markers of *il10ra* mutant macrophages in the scRNA-seq time course. **(a)** Schematics of neomycin regime and time point collection for scRNA-seq. (**b**) Integrated UMAP of the 6 datasets. (**c**) Feature plots of cluster marker genes.

Supplementary Figure 9

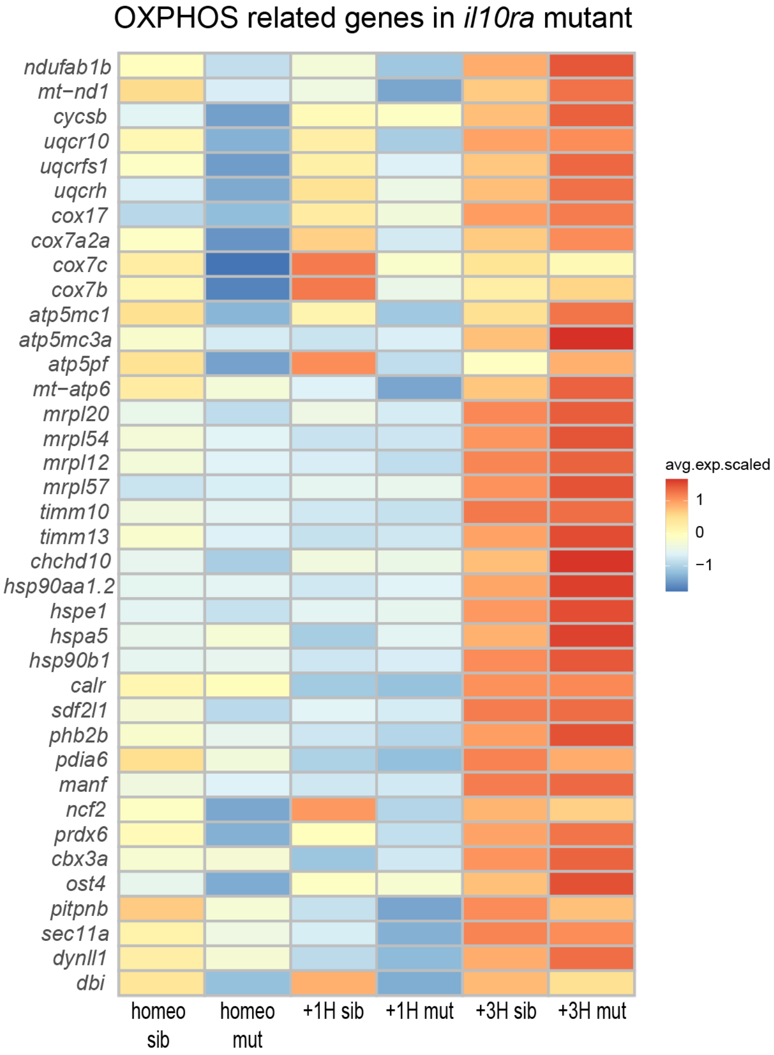

Supplementary Figure 9. Induction of oxidative phosphorylation related genes is not affected in the *il10ra* mutant. Heatmap for oxidative phosphorylation related genes at each time point between *il10ra* mutants and siblings from the scRNA-seq datasets.

Supplementary Figure 10

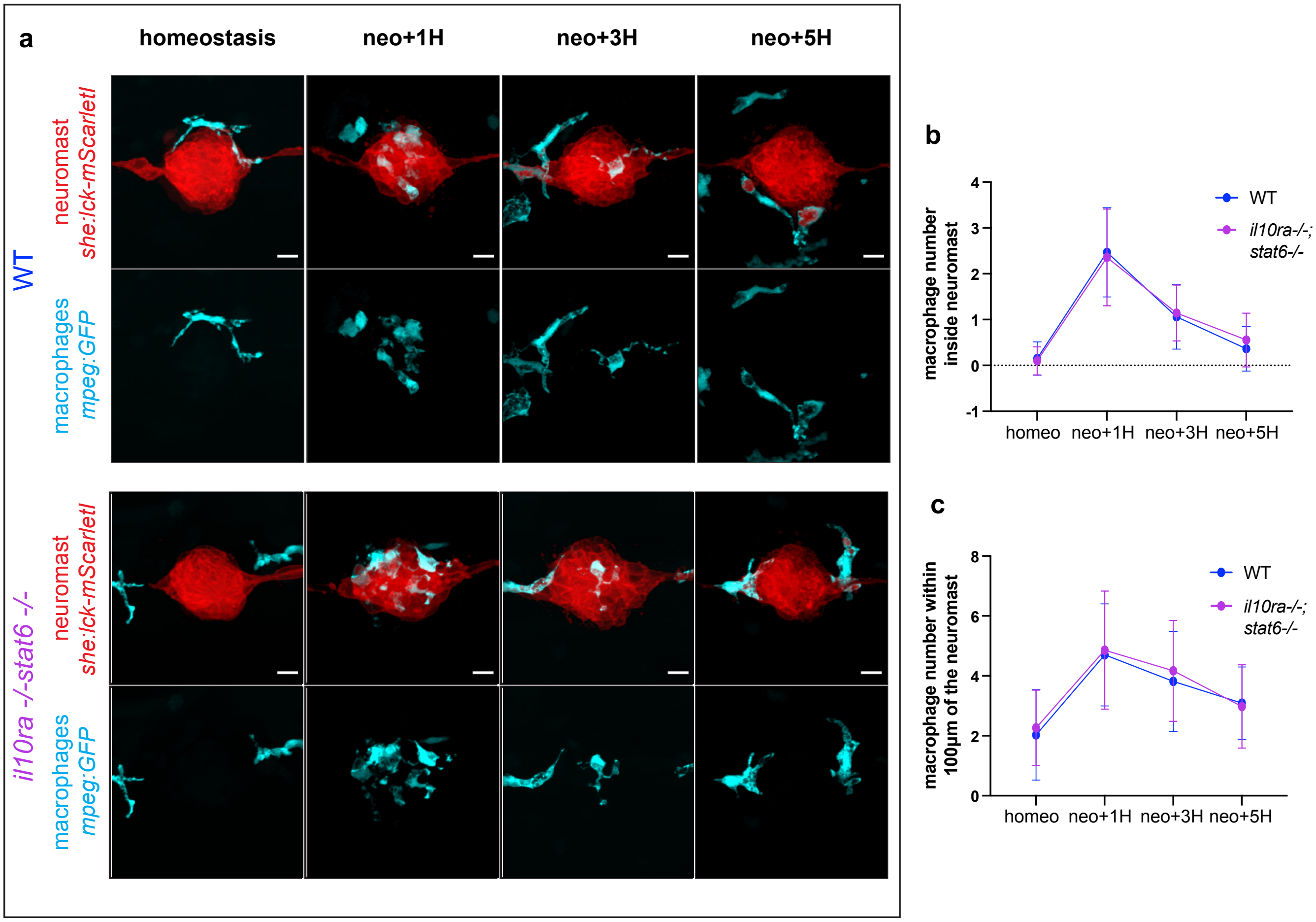

Supplementary Figure 10. Loss of IL10 and IL4 signaling does not affect macrophage dynamics after HC death. (a) Representative confocal images (projection of a 30mm z-stack) of the macrophage recruitment assay. (**b-c**) Quantification of macrophages in (b) or around (c) the neuromast. Each dot represents the number of macrophages per neuromast (3 neuromasts per larvae, 11-15 larvae per condition and 3 biological replicates). For all graphs, data are represented as mean +/- SD.
