# Supplementary material for: An anti-inflammatory activation sequence governs macrophage transcriptional dynamics during tissue injury in zebrafish
Source: Nat Commun. 2022 Sep 20;13:5356. doi: 10.1038/s41467-022-33015-3 (PMC9489698; doi:10.1038/s41467-022-33015-3)
Supplement: Supplementary file 3 — Description of Additional Supplementary Files [file 41467_2022_33015_MOESM3_ESM.pdf]

**Supplementary Data 1: "Cluster markers of mpeg:GFP cells from the integrated macrophage time course dataset".** This spreadsheet contains the differentially expressed gene list (cluster markers) of mpeg:GFP cells from the integrated macrophage time course dataset generated with Seurat::FindAllMarkers() with default parameters. Genes with p-values greater than 0.05 were omitted from the gene list. Each tab represents one cluster.

**Supplementary Data 2: "Differentially expressed genes for each time point for the effector macrophages population".** Each tab represents the Differentially Expressed genes shared by the three effector clusters based on the Venn Diagram.
Tabs are separated by time point and up- or downregulated genes
Each tab contains a GO and pathway enrichment analysis using Metascape.

**Supplementary Data 3:**
**Title "Cluster markers of mpeg:GFP cells from the integrated il10ra mutant macrophage time course dataset".** This spreadsheet contains the differentially expressed gene list (cluster markers) of mpeg:GFP cell from the integrated il10ra mutant macrophage time course dataset generated with Seurat::FindAllMarkers() with default parameters. Genes with p-values greater than 0.05 were omitted from the gene list. Each tab represents one cluster.

**Supplementary Movie 1. 3D animation of a whole 5dpf larva, related to Figure 1a.** Macrophages (*mpeg:GFP*) are labelled in cyan and neuromasts (*she:lckmScarletI*) in red.

**Supplementary Movie 2. Effector macrophages rapidly phagocytose dying HCs upon addition of neomycin.** Maximum projection (z-stack of 30□m) of a time-lapse recording of macrophages (blue, *mpeg*:*GFP*) and HCs (red, *myo6*:lckmScarletI). One image is taken every minute for two hours.

**Supplementary Movie 3. Zoom of macrophages phagocytosing HCs from Supplementary Movie 2**

**Supplementary Movie 4. Dynamics of macrophages during 7H after neomycin treatment related to Figure1b.** Maximum projection (z-stack of 30□m) of a time lapse recording of macrophages (cyan, *mpeg*:*GFP*) and neuromasts (red, *she*:*lckmScarletI).* One image is taken every five minutes for seven hours.

**Supplementary Movie 5. Effector macrophages are located close to the neuromasts, and all macrophages increase their velocity upon neomycin treatment.** Maximum projection (z-stack of 90mm) of a time lapse recording of macrophages (cyan, *mpeg*:*GFP*) and neuromasts (red, *she*:*lckmScarletI*) in the trunk region of 5dpf larvae. One image is taken every minute for two hours.
