# Supplementary material for: An anti-inflammatory activation sequence governs macrophage transcriptional dynamics during tissue injury in zebrafish
Source: Nat Commun. 2022 Sep 20;13:5356. doi: 10.1038/s41467-022-33015-3 (PMC9489698; doi:10.1038/s41467-022-33015-3)
Supplement: Supplementary file 12 — Reporting Summary [file 41467_2022_33015_MOESM12_ESM.pdf]

nature research

Corresponding author(s): Tatjana Piotrowski

Last updated by author(s): Jul 25, 2022

# Reporting Summary

Nature Research wishes to improve the reproducibility of the work that we publish. This form provides structure for consistency and transparency in reporting. For further information on Nature Research policies, see our Editorial Policies and the Editorial Policy Checklist.

## Statistics

For all statistical analyses, confirm that the following items are present in the figure legend, table legend, main text, or Methods section.

| n/a | Confirmed | |
|---|---|---|
| ☐ | ☒ | The exact sample size (*n*) for each experimental group/condition, given as a discrete number and unit of measurement |
| ☒ | ☐ | A statement on whether measurements were taken from distinct samples or whether the same sample was measured repeatedly |
| ☐ | ☒ | The statistical test(s) used AND whether they are one- or two-sided<br>*Only common tests should be described solely by name; describe more complex techniques in the Methods section.* |
| ☒ | ☐ | A description of all covariates tested |
| ☒ | ☐ | A description of any assumptions or corrections, such as tests of normality and adjustment for multiple comparisons |
| ☐ | ☒ | A full description of the statistical parameters including central tendency (e.g. means) or other basic estimates (e.g. regression coefficient) AND variation (e.g. standard deviation) or associated estimates of uncertainty (e.g. confidence intervals) |
| ☐ | ☒ | For null hypothesis testing, the test statistic (e.g. *F*, *t*, *r*) with confidence intervals, effect sizes, degrees of freedom and *P* value noted<br>*Give P values as exact values whenever suitable.* |
| ☒ | ☐ | For Bayesian analysis, information on the choice of priors and Markov chain Monte Carlo settings |
| ☒ | ☐ | For hierarchical and complex designs, identification of the appropriate level for tests and full reporting of outcomes |
| ☒ | ☐ | Estimates of effect sizes (e.g. Cohen's *d*, Pearson's *r*), indicating how they were calculated |

*Our web collection on statistics for biologists contains articles on many of the points above.*

## Software and code

Policy information about availability of computer code

| | |
|---|---|
| Data collection | Nikon Elements AR 4.6 (Nikon) software for confocal microscopy aquisition |
| Data analysis | Fiji (version 2.3.0) and Imaris (version 9.0 and 9.6) were used for all image analysis and quantification.<br><br>R (version 3.6.3) and Seurat (v3.2.0) were used for single-cell RNA sequencing analysis.<br><br>GraphPad Prism 8 (version 8.4.3) was used for statistical analysis. |

For manuscripts utilizing custom algorithms or software that are central to the research but not yet described in published literature, software must be made available to editors and reviewers. We strongly encourage code deposition in a community repository (e.g. GitHub). See the Nature Research guidelines for submitting code & software for further information.

## Data

Policy information about availability of data

All manuscripts must include a data availability statement. This statement should provide the following information, where applicable:

- Accession codes, unique identifiers, or web links for publicly available datasets
- A list of figures that have associated raw data
- A description of any restrictions on data availability

Original data used for the results reported in this paper may be accessed from the Stowers Original Data Repository at http://www.stowers.org/research/publications/libpb-1663. Raw and processed scRNAseq data generated in this study have been deposited in the Gene Expression Omnibus database (#GSE209884; https://www.ncbi.nlm.nih.gov/geo/query/acc.cgi?acc=GSE209884)

# Field-specific reporting

Please select the one below that is the best fit for your research. If you are not sure, read the appropriate sections before making your selection.

☒ Life sciences ☐ Behavioural & social sciences ☐ Ecological, evolutionary & environmental sciences

For a reference copy of the document with all sections, see nature.com/documents/nr-reporting-summary-flat.pdf

# Life sciences study design

All studies must disclose on these points even when the disclosure is negative.

| | |
|---|---|
| Sample size | No statistical analysis was used to calculate sample size. Sample size was determined based on consensus practice in the field |
| Data exclusions | No data were excluded from analysis |
| Replication | All attempts at replication were successful. All experiments were repeated at least three independent times (with multiple measures/animals per experiment) to confirm reproducibility. Each scRNAseq time point represent one dataset without replicates (biological or technical) following the consensus in the field. |
| Randomization | the scRNAseq datasets where downsampled randomly to 14000 cells to normalize cells samples across time points |
| Blinding | No blinding was required. Data were reproduced and analyzed multiple times to ensure robustness. |

# Reporting for specific materials, systems and methods

We require information from authors about some types of materials, experimental systems and methods used in many studies. Here, indicate whether each material, system or method listed is relevant to your study. If you are not sure if a list item applies to your research, read the appropriate section before selecting a response.

## Materials & experimental systems

| n/a | Involved in the study | |
|---|---|---|
| ☐ | ☒ | Antibodies |
| ☒ | ☐ | Eukaryotic cell lines |
| ☒ | ☐ | Palaeontology and archaeology |
| ☐ | ☒ | Animals and other organisms |
| ☒ | ☐ | Human research participants |
| ☒ | ☐ | Clinical data |
| ☒ | ☐ | Dual use research of concern |

## Methods

| n/a | Involved in the study | |
|---|---|---|
| ☒ | ☐ | ChIP-seq |
| ☒ | ☐ | Flow cytometry |
| ☒ | ☐ | MRI-based neuroimaging |

## Antibodies

| | |
|---|---|
| Antibodies used | Primary antibodies: HCS-1 (otoferlin from Hybridoma Banks) 1/500 dilution; VAMP2 (Genetex, #GTX132130) 1/500 dilution. |
| Validation | All these antibodies are commercially available and were published in peer reviewed articles. Antibody were validated by assessing its positivity in tissues known to express these proteins (hair cells for HCS-1 and synapses for VAMP2). |

## Animals and other organisms

Policy information about studies involving animals; ARRIVE guidelines recommended for reporting animal research

| | |
|---|---|
| Laboratory animals | All experiments were performed following the guidelines from the Stowers Institute IACUC review board. Experiments were performed on zebrafish larvae at 5 to 7 dpf. Sex cannot be determined at this stage. |
| Wild animals | This study did not involve wild animals |
| Field-collected samples | This study did not involve samples collected from the field |
| Ethics oversight | This study was conducted in accordance with the Guide of the Care and Use of Laboratory Animals of the National Institute of Health and protocols were approved by the Institutional Animal Care and Use Committees of the Stowers Institute for Medical Research (Sensory lateral line development and hair cell regeneration in zebrafish, TP Protocol: # 2017-0176) |

Note that full information on the approval of the study protocol must also be provided in the manuscript.
